# Linking knowledge and attitudes: Determining neurotypical knowledge about and attitudes towards autism

**DOI:** 10.1371/journal.pone.0220197

**Published:** 2019-07-25

**Authors:** Rebecca Kuzminski, Julie Netto, Joel Wilson, Torbjorn Falkmer, Angela Chamberlain, Marita Falkmer

**Affiliations:** 1 School of Occupational Therapy, Social Work and Speech Pathology, Curtin University, Perth, Western Australia, Australia; 2 Cooperative Research Centre for Living with autism (Autism CRC), Long Pocket Brisbane, Queensland, Australia; 3 Pain and rehabilitation Centre, department of Medical and Health Sciences, Linkӧping University, Linkӧping, Sweden; 4 School of Education and Communication, CHILD programme, institute of Disability Research, Jӧnkӧping University, Jӧnkӧping County, Sweden; University of New South Wales, AUSTRALIA

## Abstract

“Why are neurotypicals so pig-ignorant about autism?” an autistic person wrote on the Curtin Autism Research Group’s on-line portal as a response to a call for research questions. Co-produced with an autistic researcher, knowledge about and attitudes towards autism were analysed from 1,054 completed surveys, representing the Australian neurotypical adult population. The majority, 81.5% of participants had a high level of knowledge and 81.3% of participants had a strong positive attitude towards autism. Neither age, nor education level had an impact on attitudes. However, attitudes were influenced by knowledge about ‘Societal Views and Ideas’; ‘What it Could be Like to Have Autism’; and the demographic variables ‘Knowing and having spent time around someone with autism’; and gender (women having more positive attitudes than men). Thus, targeted interventions, geared more towards men than women, to increase knowledge about autism could further improve attitudes and increase acceptance of the autistic community.

## Introduction

### Terminology

Autism spectrum disorder (ASD) is a neurodevelopmental condition characterised by social and communication deficits, and repetitive and restricted activities, behaviours and interests, causing social and occupational impairment [1]. While the term ASD will be used regarding the diagnostic condition, ‘autistic person/people’ will be used where possible, when not referring to a source that cannot be changed for copyright reasons such as the Societal Attitudes Towards Autism scale. This preferred language selection was made in consultation with autistic people, including one of the study originators. Accordingly, the terms, ‘autistic community’ and ‘autism community’ will also be used throughout. ‘Autistic community’ describes the community of autistic people, whereas ‘autism community’ describes the community of people affected by autism, inclusive of autistic people, their families, friends, and service providers. This choice of terminology is also underpinned by a recent survey conducted in the United Kingdom about preferred language, that found 61% of adults with a diagnosis of ASD, 52% of family members and friends, and 51% of parents endorsed the use of ‘autistic’ [2]. While this obviously may not be the preferred language of every individual on the autism spectrum, family member or friend, this is the preference of terminology used in the present study. Where suitable, people without autism or any other neurological condition will be labelled as ‘neurotypical’. Co-production in the context of this study refers to the project being produced in collaboration with an autistic peer that has been involved from the initiation of the project and throughout the whole research process. Furthermore, consultations with members of the Australian autistic community have been conducted during the process.

### Background

The present study was conducted in Australia where the incidence of ASD is rising, with a 41.2% increase seen between 2012 and 2015 [3]. The estimated number of cases climbed from 115,400 in 2012, to 164,000 in 2015 [3]. Only 40.8% of the autistic community were reported to participate in the workforce, with the majority experiencing unemployment and social isolation [3], despite expressing desire to be employed, develop social awareness, friendships and romantic relationships [4]. Indeed, autistic adolescents have fewer close friendships, receive fewer social phone calls, and are less commonly invited to engage in social activities, when compared to people who experience intellectual disability, learning disability or “emotional disturbance” despite access to the same educational support services [5].

Being treated differently to neurotypicals, either harshly or cautiously, results in autistic people feeling ‘different’ while trying to be ‘normal’ adults [6]. Higher education is reportedly particularly challenging for autistic people, due to the interpersonal communication skills often required [7]. Participation in higher education may be additionally challenging for autistic people because of attitudes of peers without autism, which may lead to further social isolation [7]. Higher education is seen to correlate with higher income and increased quality of life, therefore, if attitudes of peers are deterring autistic people from pursuing higher education this could further decrease their quality of life [7]. In fact, quality of life of autistic people has been reported as lower compared to those without autism throughout the lifespan [8].

The experiences and challenges discussed above, in the narratives of the autistic and autism communities, may be a result of stigma. Stigma comprises of three key elements; knowledge issues (ignorance); attitudinal issues (prejudice), and behavioural issues (discrimination) [9]. Consequently, knowledge and attitudes in the neurotypical population may contribute to how autistic people are treated.

In contrast to the research question *“Why are neurotypicals so pig-ignorant about autism*?*”* posted on the Curtin Autism Research Group’s on-line portal as a response to a call from the autistic community for research questions, previous research suggests that neurotypical adults feel somewhat comfortable around autistic people [9]. While uncertain about the cause of autism but believing it was beyond the person’s control, popular perception is that autistic people have exceptional abilities in areas such as art and IT, in contrast to their limited functional skills for independence and inability to fully express themselves [10]. However, portrayals of autistic people in the media may influence neurotypicals’ beliefs (knowledge) about and attitudes towards autism [10]. In fact, a study conducted with college students reported that knowledge was relatively easy to change compared to stigma, which is also based on attitudes [11]. Furthermore, research in children concluded that increased awareness of autism could lead to increased positive attitudes and behaviours towards children with autism [12]. It is, therefore, important to know what knowledge the neurotypical population has about autism and how this may influence their attitudes towards autistic people.

The few existing studies regarding attitudes toward autism report inconclusive results, with small and non-representative populations [9, 10]. To date, no co-produced study exists where knowledge about autism is defined from the viewpoint of the autistic community, nor are any studies on the topic conducted in the Australian context.

Consequently, this study aimed to investigate neurotypicals’ knowledge about autism, explore possible relationships between this knowledge and their attitudes towards autism, and, finally, identify demographic factors that may further influence attitudes. If successfully investigated, best targeted future interventions in the broader community could be determined.

## Methods

### Design

A co-produced cross-sectional design was used to survey an array of the Australian neurotypical adult population. Data were collected through an online survey. Throughout the duration of this study, autistic people and the autism community have contributed and continuously been consulted for their expertise. This collaboration included the generation of the research question by an autistic co-researcher, who remained involved throughout the project. The autistic community who engage in the Curtin Autism Research Group social media pages were asked to provide questions they would like to be asked in a knowledge questionnaire. Following this, the autistic co-researcher collaboratively selected, refined, grouped and labelled the questions for a knowledge questionnaire. The autistic co-researcher also assisted with interpretation of results, the writing of this article, and advised the selected language choice used throughout this article.

### Participants

Participants were recruited using purposive, convenience and snowball sampling techniques through social media such as Facebook and newsletters, and personal contact in public places and via organisations, such as sports clubs. Recruitment was further aided by a Curtin University Media Release in Western Australia, which resulted in a Western Australian radio interview and an Australia-wide electronic news article published on two news websites. To be eligible for participation, people had to report being over 18 years of age, residents of Australia, have sufficient English, and report no ASD or other cognitive diagnosis. As part of the consent to participate, participants self-reported that they met the inclusion and exclusion criteria provided in the participant information. To further safeguard against those under 18 years completing the survey, any entry with reported age below 18 was directed to the end of the survey preventing completion of any further questions. These submissions were, subsequently, excluded from the study.

To obtain a large representative sample, the survey had to be easy to access and quick to finalise. As the survey was promoted electronically on social media via a link posted on community and personal pages, participants could access the survey from their own devices. Participants who were sought through personal contact at public places either completed the survey online using an iPad, or on a paper copy of the survey form. The survey was administered between March and August 2017 and took on average less than 10 minutes to complete.

A total of 1,150 entries were submitted. Of these, 1,078 entries completed the standardised Societal Attitudes Towards Autism (SATA) section, met the inclusion/exclusion criteria, and were used for analysis. Only 1,054 entries were considered in the co-produced knowledge section and further statistical analyses, as 24 participants had not begun the knowledge section. Participants across all six states of Australia were sampled, including metropolitan (metro) and regional areas. Most participants were highly educated, as shown in Table 1.

**Table 1:** Participant demographics.

Demographic	Participants	
	n	%
Gender		
Male	237	22.0
Female	837	77.6
Other	4	0.4
Age		
18–29	247	22.9
30–39	253	23.5
40–49	289	26.8
50–59	160	14.8
60–69	79	7.3
70–79	32	3.0
80–89	2	0.2
Unreported	16	1.5
Education		
Primary	1	0.1
High school/TAFE*/Apprentice	453	42.0
Undergraduate	329	30.5
Postgraduate	295	27.4
State (AUS)		
Australian Capital Territory	17	1.6
New South Wales	59	5.5
Northern Territory	2	0.2
Queensland	76	7.1
South Australia	35	3.2
Tasmania	4	0.4
Victoria	49	4.5
Western Australia	836	77.6
Region		
Metro	891	82.7
Regional	187	17.3
Know/have experience with autistic people		
Yes	823	76.3
No	255	23.7
Total (people)	1,078	100

*Technical and Further Education

T-tests concluded that male participants were on average 3.82 years older (*SE* = 1.039) than female participants (*p =* 0.007). Furthermore, participants living in regional areas were on average 2 years older (*SE* = 0.497) than those living in metro areas (*p =* 0.034). Mann Whitney U tests were conducted for gender and all education levels, which found no significant differences between the groups (*p =* 0.452, *p =* 0.086, *p =* 0.368). A Chi Square test was conducted for gender and location (metro/regional), which found no significant differences between these groups (*p =* 0.781). To assess the relationship between age and education a Spearman’s correlation was conducted, which found no significant correlation (*p =* 0.447).

The population recruited during data collection was not a perfect fit to the Australian population demographics. Far more participants were sampled in Western Australia (WA), despite the vast majority of the population residing in the eastern states of New South Wales and Victoria [13]. This could be attributed to the research originating from WA, therefore skewing the sample because of the accessibility of participants in WA. The Australian population has a 50.7% representation of females and 49.3% representation of males [13]. As Table 1 presents, the number of females was more than twice the number of males in this sample. The median age of the Australian Population is 37.2 years, whereas the censored population in Table 1 (i.e., no one under the age of 18 years) had a median age of 41 years [13]. The sample gathered in this research was representative of more people living in metro areas than that of the general population, in which only two thirds of the population live in metro areas [13]. It can also be noted that this sample population had a larger proportion of higher educated people than that of the Australian population. Only 17% of the Australian population report attainment of an undergraduate degree, 2.8% attainment of a graduate diploma or certificate, and 5.5% a post graduate degree [14].

### Materials

The co-produced survey consisted of 3 sections; demographics, attitudes and knowledge. The demographics section included non-identifying questions about the person completing the survey. Tables 2 and 3 present all the questions in the survey.

**Table 2:** Demographics and SATA questions.

Demographics
Do you know and have you spent time around someone with autism?
What is your age?
What is your gender
What is your highest level of education?
What state or territory do you live in?
What region do you live in?
Societal Attitudes Towards Autism Scale
People with autism should not engage in romantic relationships.
* People with autism should have the opportunity to go to university.
People with autism should not have children.
People with autism should be institutionalised for their safety and others.
If a facility to treat people with autism opened in my community, I would consider moving out.
Individuals with autism are incapable of living on their own.
I would be afraid to be around a person with autism.
A person with autism is an emotional burden to his/her family.
* I would be comfortable sitting next to a person with autism in the same class.
A person with autism is a financial burden to his/her family.
People with autism should be encouraged to marry a person with autism.
People with autism are incapable of forming relationships and expressing affection.
* Children with autism should be fully integrated into mainstream classes.
I would be uncomfortable hugging a person with autism.
People with autism cannot understand other people’s feelings.

* = reverse scored

**Table 3:** Co-produced knowledge question sections for analysis. The order of the statements was mixed across sections in the survey.

	Correct answer
Section 1: **Societal Views and Ideas**	
The character “Sheldon” in Big Bang Theory is an accurate portrayal of an autistic person.	S
Movies such as “Rainman” show an accurate depiction of all autistic people.	N
Autistic people are eternally childlike.	N
Autism is described in the news in a balanced way.	N
You can tell a person is autistic by looking at them.	N
Vaccines do not cause autism.	T
Section 2: **What it Could be Like to Have Autism**	
Autistic people can choose what behaviours they display in public.	S
Autistic people experience emotions differently to people who do not have autism.	N
Autistic people do not understand sarcasm in jokes and can take them literally.	S
Autistic people can learn to become less sensitive to stimuli, such as noise, touch and smell.	N
Autistic people have difficulty understanding what to do in social situations.	T
Autism is a stable condition that does not vary day to day.	N
Section 3: **Characteristics of Autism**	
* Autism never occurs in adults.	N
Autistic people are sensitive to certain stimuli, such as noise, touch and smell.	S
All autistic people have intellectual disability.	N
Autism has a range of symptoms and severities.	T
* Autism only occurs in children.	N
All autistic people have a genius level of intellect.	N
Section 4: **Strengths and Challenges of Having Autism**	
All autistic people have the same strengths.	N
All autistic people have the same challenges.	N
The level of support required by autistic people does not change with age.	N
Autistic people can be employed in competitive paying jobs.	T

* = used for testing internal consistency, S = somewhat true, T = true, N = not true

The standardised SATA scale [15] measured the attitudes towards autism. This 16-item measurement for societal attitudes towards autism was developed in the United States of America. Originally, the scale was used with college students, however the present study used the scale with the general Australian neurotypical population. The SATA scale has reported a level of internal consistency of 0.86 [15]. It was obtained on-line, where it was available for public use. After gaining permission via correspondence with the author of the SATA scale, terminology changes were made to ensure the tool was culturally appropriate to the Australian adult population, such as changing “college” to “university”. Question 16, the 3^rd^ school-based question of the SATA, was not applicable to adults and therefore it was removed. The SATA used in the current study therefore comprised of 15 questions. Each question was scored on a 4-point Likert scale from strongly disagree (1) to strongly agree (4) [15].

At the time of the current study, one validated measure assessing knowledge about autism was found [16]. In order to stay true to the intention of co-produced research, this measure was assessed and deemed as having a too narrow focus. Consequently, a set of questions assessing knowledge areas perceived as relevant was developed in consultation with the autistic community, forming a knowledge section added to the survey. Via a social media platform autistic people and their families were asked about common misconception they met from the neurotypical population, and what questions they thought would be relevant in assessing knowledge about autism in this population. Neurotypical people were also given an opportunity to share questions they wished to ask an autistic person. All de-identified responses were subsequently used to co-produce the knowledge questions. The initial ‘short list’ of questions was altered following consultation with a reference group of autistic people. Before using the final set of questions, a researcher with a background in psychology reviewed the questions, advising changes in order to limit the impact of the phrasing shaping participants’ responses. The developed knowledge section of the questionnaire comprised 22 questions. Scoring was awarded using a 4-point Likert scale with response options of “true”, “somewhat true”, “I don’t know” and “not true”. In accordance with the co-produced set of questions, answers were awarded a score for how correct they were, with 0 = incorrect, 1 = I don’t know; 2 = somewhat correct; and 3 = correct. The scoring of this section of the questionnaire was established in close consultation with autistic people, who deemed higher scores awarded for ‘I don’t know’ than actually being wrong as very important. The “I don’t know” option was included as indicated lack of information/knowledge in the specific area of the question. Table 3 presents the knowledge questions as per groupings for analysis.

### Pilot testing

The survey was piloted with 7 participants who represented various demographics in the population to assess its usability (Table 4). These participants were gathered through personal contact, and all completed the survey via the electronic link on multiple devices, such as smart phones, desk top and lap top computers. These participants did not suggest any changes, therefore data collection began.

**Table 4:** Pilot group.

Person	Gender	Age	Education	Know person with ASD^1^	State (AUS)	Region
1	M	20	UG	Yes	WA	Metro
2	F	48	HS	Yes	WA	Metro
3	M	49	UG	No	WA	Metro
4	M	53	UG	No	WA	Metro
5	M	72	HS	Yes	WA	Metro
6	F	18	HS	Yes	WA	Metro
7	F	70	HS	Yes	WA	Metro

M = male, F = female, HS = High school/Technical and Further Education /Apprentice, UG = Undergraduate, WA = Western Australia

^1^The full extent of the question was: “Do you know and have you spent time around someone with autism?”

### Analysis

Survey data were entered into IBM Statistical Package for the Social Sciences (SPSS) version 23 [17]. Demographic frequencies were determined and Kolmogorov Smirnov testing for normal distribution of responses was undertaken, with no demographic data presented as normally distributed. The demographic data were, however, very close to normally distributed when observed in graphical format of expected over observed values. Non-parametric tests were undertaken to determine the significance of demographic information, as previously presented.

The SATA scores were totalled (maximum score = 60) for each participant, with high scores representing a positive attitude. Questions from the knowledge section of the questionnaire were firstly summed in total, then grouped into four sections. These sections (Table 3) were co-produced with autistic people. Each section’s scores were then summed. The knowledge section of the survey obtained a Cronbach’s alpha of 0.76, demonstrating an adequate level of internal consistency. Whilst the general cut-off to conclude a survey has optimal internal consistency of 0.8, the minimum for a tool deemed appropriate for use is 0.7 [18].

Following this, backwards entry multiple linear regression modelling was conducted, to determine independent variables that influenced SATA scores, representing attitudes towards autism. All assumptions of the multiple linear regression were met, as The Central Limit Theorem assumes that with large sample sizes like this, a good representation of a normal distribution is provided [19].

### Ethics

All participants provided consent by choosing to agree with the information about the project prior to beginning the survey, and no identifying data were collected. All data were input into the Qualtrics survey platform, then downloaded and stored electronically on a password protected drive. All paper surveys were kept in a locked cupboard in a locked office on Curtin University property in Western Australia. The study was approved by the Human Research Ethics Committee at Curtin University, Perth, Western Australia (approval number HRE2016-0409).

## Results

### Attitudes towards autism

Summing the total SATA score for each participant determined if each had a positive or negative attitude towards autism. Total scores were separated into four categories; scores of 15 or less showed a strong negative attitude, 16–30 showed a negative attitude, 31–45 showed a positive attitude, and 46–60 showed a strong positive attitude [15]. All but one participant had a positive attitude towards autism. A much larger proportion, 81.3% of the overall population, reported a strong positive attitude towards autism (Table 5). The mean score of the SATA section of the questionnaire was 54 (*SE* = 0.164). The maximum score, 60, was obtained by 29 participants. The minimum score of any participant was 24.

**Table 5:** Overall attitudes towards autism.

Attitude towards Autism	n	%
Strong Negative	0	0.0
Negative	1	0.1
Positive	201	18.6
Strong Positive	876	81.3
Total	1078	100.0

### Knowledge about autism

The co-produced knowledge section of the survey had, as mentioned, 1,054 entries. The mean total knowledge score was 51.3 (*SE* = 0.214). The median score was 53, being in the high range. The maximum score obtained by a participant was 65, and the minimum 21. Looking at the participants’ total knowledge scores, 81.5% of this population had a high level of knowledge (Table 6).

**Table 6:** Knowledge total scores.

Knowledge Level	Score	n	%
Low	(0–22)	3	0.3
Medium	(23–44)	192	18.2
High	(45–66)	859	81.5

To gain further understanding of which areas of knowledge this population knew more about, the survey was split into four sections and compared (Table 7). It was found that many participants scored high in the *Societal Views and Ideas*; *Characteristics of Autism*; and *Strengths and Challenges* sections. Each had over 70% of the population scoring in the high range. However, the participants scored lower in section two: *What it Could be Like to Have Autism*, with 55.5% of the population scoring in the mid-range. Knowledge about section three; *Characteristics of Autism*, had the highest proportion of high knowledge scores compared to all other sections. *Characteristics of Autism* also had the second lowest proportion of “I don’t know” responses. Interestingly, the highest proportion of “I don’t know” responses was reported in the *Societal Views and Ideas* section, having an average of 20.2% of “I don’t know” responses per question. The single question with the highest “I don’t know” response rate also occurred in this section, being the question; “The character ‘Sheldon’ in ‘Big Bang Theory’ is an accurate portrayal of an autistic person”. The second highest average proportion of “I don’t know” responses was the section *What it Could be Like to Have Autism*, which had an average of 17.5% “I don’t know” responses per question.

**Table 7:** Knowledge section scores.

Knowledge Section (score range)	Mean score (SD)	n	%
1: Societal Views and Ideas (4–18)	13.9 (2.8)		
Knowledge Level			
Low (0–6)		12	1.1
Medium (7–12)		291	27.6
High (13–18)		751	71.3
2: What it could be Like to Have Autism (3–18)	11.4 (2.8)		
Knowledge Level			
Low (0–6)		48	4.6
Medium (7–12)		585	55.5
High (13–18)		421	39.9
3: Characteristics of Autism (6–18)	15.5 (2.6)		
Knowledge Level			
Low (0–6)		7	0.7
Medium (7–12)		130	12.3
High (13–18)		917	87.0
4: Strengths and Challenges (2–12)	10.5 (1.9)		
Knowledge Level			
Low (0–4)		20	1.9
Medium (5–8)		122	11.6
High (9–12)		912	86.5

### Determining influential factors affecting attitudes

The sum of the SATA scores was set as the dependent variable for two backwards multiple linear regression models. The independent variables included all demographic questions (except state location), the sum of the total knowledge score in the first analysis and the sum of each knowledge section in the second analysis. Independent variables were removed based on their contribution to the model as indicated by their p-values.

The model generated from the first backwards multiple linear regression, presented in Table 8, had an *r*^*2*^ value of 0.129. This model showed no perfect co-linearity, as no Pearson’s correlation values were above 0.9, suggesting that minimal to no multicollinearity was occurring within the data [19]. Tolerance values were above 0.2 and close to 1.0, further supporting that minimal to no multicollinearity was occurring in the data [19]. Finally, VIF were well below 10 and close to 1 suggesting minimal to null impact of multicollinearity on the data. The data were also checked for independent error using a Durbin-Watson (DW) test. This model obtained a DW score of 1.997, inferring no independent error, i.e., the residuals of the model were uncorrelated. Further statistical testing of the presence of confounders and effect modifiers were not deemed necessary based on these results.

**Table 8:** Backward multiple linear regression statistics- knowledge total score.

Model Items	*β*	Standardised *β*	*β* 95% CI	p
Do you know and have you spent time around someone with autism?	1.440	0.115	2.187–0.693	0.001
Gender	0.832	0.065	0.088–1.576	0.028
Highest level of Education	0.377	0.058	0.004–0.751	0.048
Knowledge Total Score	0.196	0.289	0.154–0.238	0.001

The second multiple linear regression analysis was conducted to scrutinise which specific areas of knowledge may have had influence on attitudes, thus, the four knowledge sections were entered into the model. The model that was generated was found to have an *r*^*2*^ of 0.133 (Table 9). Although age was not found to be significant, it was included in this model as there was a difference in age between genders.

**Table 9:** Backward multiple linear regression statistics—knowledge section scores.

Model Items	*β*	Standardised *β*	*β* 95% CI	p
Societal Views and Ideas	0.389	0.205	0.268–0.510	0.001
What Could it be like to Have Autism	0.228	0.121	0.109–0.346	0.001
Do you know and have you spent time around someone with autism?	1.443	0.115	0.697–2.188	0.001
Age	0.020	0.056	0.000–0.041	0.053
Gender	0.908	0.071	0.167–1.649	0.016

It could be noted that in this model, highest level of education was not found to be a significantly contributing variable, whereas age was a contributor very close to being a significant variable. Age is therefore presented in Table 9 for that reason.

**Fig 1:**
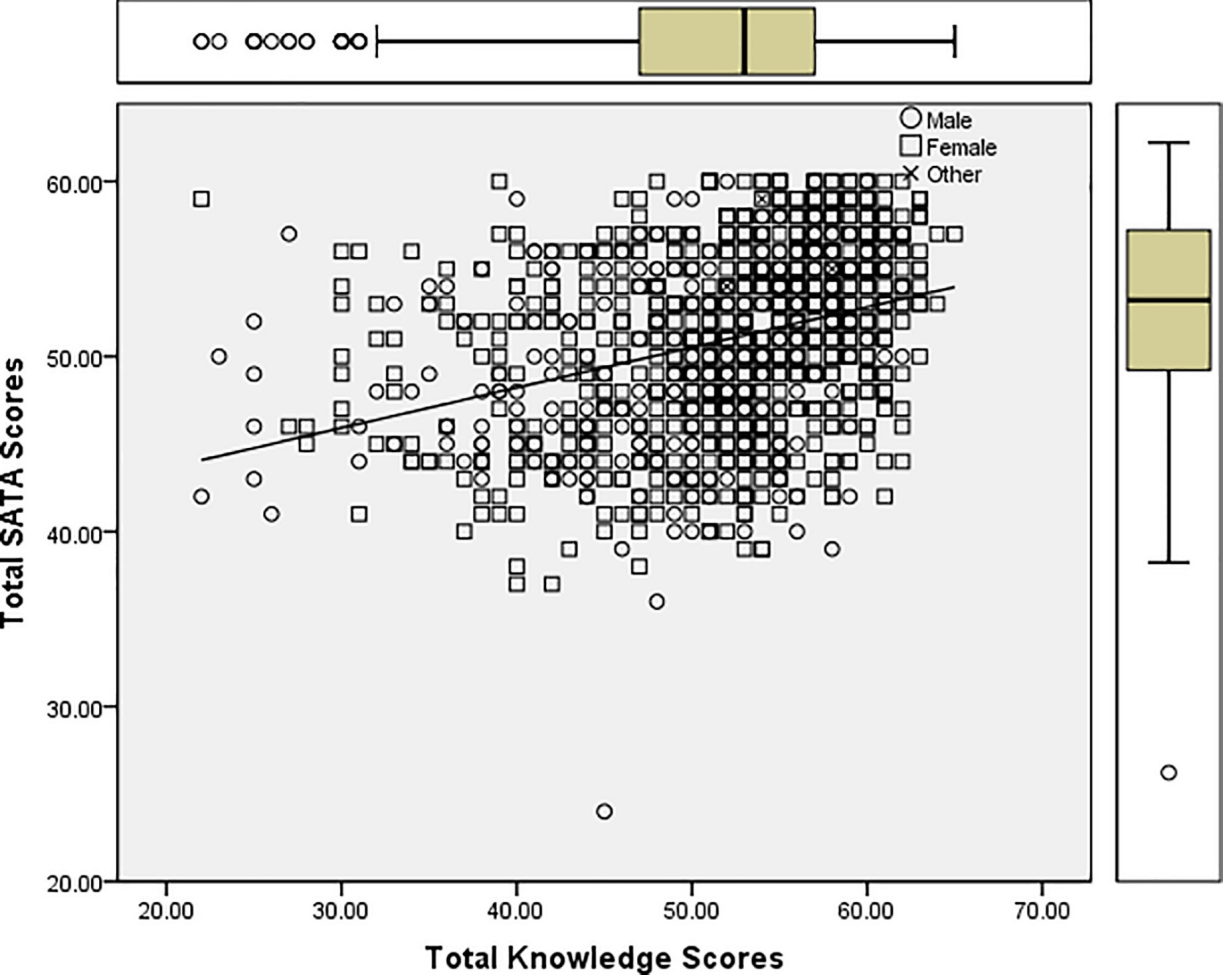
Simple scatter plot with fit line of total SATA scores by total knowledge scores by gender.

As gender was found to be a significant contributor to attitudes in the linear regression model, independent t-tests were applied, confirming a significant gender difference in the mean SATA scores with female respondents demonstrating more positive attitudes towards autism (*M* = 51.22, *SD* = 5.22) than males (*M* = 49.24, *SD* = 5.60), *t*(1072) = -5.01, *p* < 0.01, *d =* 0.37, 95% CI [-2.75, -1.22]. Independent t-tests were also performed to determine whether significant gender differences existed in respondents’ knowledge about autism. Results showed that females scored higher in total knowledge (*M* = 52.35, *SD* = 7.26) than their male counterparts (*M* = 48.11, *SD* = 8.36), *t* (1039) = -7.52, *p* < 0.01, *d =* 0.54, 95% CI [-5.35, -3.13]. A scatter plot was created to visually assess the relationship between attitudes and knowledge according to gender (Fig 1).

## Discussion

The question “Why are neurotypicals so pig-ignorant about autism?” could perhaps best be responded by the results demonstrating that clear majority in this sample were actually not ignorant about autism. In addition, 99.9% had positive or strongly positive attitudes towards autism.

An important factor that influenced attitudes towards autism was knowing and spending time around someone with autism. This appears to be a most powerful way of improving these attitudes. In support of this finding, another study found attitudes towards autism to be positively influenced by experience with autistic people [20]. First-hand experience with autistic people could provide neurotypical adults with an opportunity to dispel any misconceptions and form their own understandings of autism.

Interestingly, rather than general education level, specific knowledge about autism was found to positively influence attitudes towards autism. Knowledge in the *Societal Views and Ideas* section was found to have the highest proportion of “I don’t know” responses, with an average of 20.2% of the population reporting “I don’t know” per question. Questions in this section were largely based on media representations of autism. This finding could result from participants not being familiar with the specific media representations that was referred to in the questions. However, it may also emphasise the need for further knowledge translation about autism, as people with direct experience and knowledge about disability in particular used this knowledge to reject questionable messages presented in news and other media [21]. Indeed, television series and movies are media that people with no experience or knowledge about disability rely on to gain information, which in turn will influence attitudes depending on how disability, such as autism, is portrayed [21]. It was also reported by the consulted autistic adults that they met misunderstandings and preconceptions influenced by the portraying of autism in media. Consequently, it is important for media creators to consider the influence, and thereby the responsibility, they have on society’s knowledge and attitudes, particularly around disability topics, such as autism. Ideally, presenting accurate narratives of autism and ensuring ‘factual’ information is presented to the broader society in the news could assist in increasing knowledge translation to the general population. It could also be beneficial for media to present additional information to clarify how accurate, factual, or generalisable representations of autism in popular media are. This could help to better inform the population.

The other specific knowledge area found to influence attitudes towards autism was *What it Could be Like to Have Autism*. This section had the lowest level of knowledge of the four sections. The study results show that 39.9% of the population scored in the high range in this section, compared to over 70% of the population scoring high in the other three sections of the questionnaire. *Knowing What it Could be Like to Have Autism* had the second highest proportion of “I don’t know” responses. Together, these findings suggest there is a need to translate knowledge about the narrative experiences of having autism to the wider society. Ideally, understanding what it could be like to have autism should be translated to the wider population by autistic people or at least done in consultation with autistic people, as they are experts in the experience of autism [22]. Informing the population about what it could be like to have autism could aid in increasing knowledge in the *Societal Views and Ideas* section of the questionnaire, and help determine whether to accept or reject representations of autism in popular media [21]. Knowledge translation in this area could, therefore, serve as a powerful tool to increase positive attitudes towards autism.

Gender was found to significantly influence attitudes towards autism, with females demonstrating more positive attitudes than males. The mean difference in attitude scores equated to roughly 3%, with a small to medium effect size. This finding was supported by another study revealing that gender can influence attitudes in disability [23]. Furthermore, examination of the mean scores in total knowledge about autism revealed a significant gender difference, such that females scored roughly 6% higher than males, with a medium effect size. A previous study investigating public perceptions and stigma around autism, schizophrenia and bipolar disorders also demonstrated that women showed greater awareness of these conditions in general and of their specific characteristics when compared to men [9]. Statistically, these findings suggest that males might be a particular focus for interventions addressing knowledge of, and attitudes towards, autism. However, the clinical relevance of such an approach is questionable upon examination of the scatter plot in Fig 1, where a large spread of attitudes and knowledge within the groups can be seen. Thus, in summary, interventions to address attitudes towards autism and knowledge about autism (as a primary driver of attitudes) should target both males and females.

Based on the final regression model it can be anticipated that increasing specific knowledge about autism will influence attitudes towards autism in a positive way. The results of the present study show highly positive attitudes and a high level of knowledge among Australian neurotypical adults. However, autistic people have reported feeling misunderstood. It is known that knowledge and attitudes may influence behaviour [9], however, it does not always translate into acceptance [24]. So, while the population could have positive attitudes and high levels of knowledge, lack of acceptance may better explain the experience of feeling misunderstood, as reported by autistic adults [25].

Targeted interventions to increase not only knowledge about autism in general, but also about autistic individuals through personal narratives may contribute to acceptance of the autistic community in the general adult population of Australia. It could also be speculated that such interventions, along with further research into behaviour of neurotypical adults towards autistic people, could improve the overall quality of life of autistic people and foster an increase in social acceptance. In turn, a decrease in social isolation could be anticipated.

### Limitations

It is important to note that this sample was close to being representative of the Australian population, but not a 100% representative sample of it. The findings of the present study should therefore be used with caution as they may not be completely generalisable to the Australian population without further research. This sample may also be impacted by bias as most reported knowing an autistic person, which could have influenced the results obtained. It is also possible that the sample represent the fact that autism is fairly common and many people have some connection to a person with autism through their personal or professional network [16]. However, 255 participants in the current study reported that they did not know any autistic person, which is not an insignificant number of participants from a statistical perspective. Future research should, however, attempt to capture a large array of people who do not know autistic people to investigate in-depth the impact of it. A higher proportion of females were also sampled, and although gender could be accounted for and over 200 participants were male, it would still be ideal for future research to strive to obtain a sample with more male participants.

The SATA scale was validated in a population of collage respondents. It is possible that the questions were phrased in a way that made it easy to deem what would be a socially desirable response, hence, the high scores may be a result of social desirability bias, indicating that the scale needs to be further developed and validated.

The current study developed a knowledge section in the questionnaire based on the reported misunderstandings and misconceptions of autistic adults and/or family members of autistic individuals, since an existing Autism Awareness Survey used in a previous study [16], was deemed too ‘narrow’ in its scope of knowledge about autism. Hence, the knowledge section of the survey in the current study was not standardised. However, it was found to have a Cronbach’s alpha of 0.76. In comparison, the reported internal consistency of the Autism Awareness Survey was 0.60 [16]. Future research should aim to conduct a complete validation of a co-produced scale knowledge scale. It is noteworthy that the grouping of questions could be done in many ways, but as this study was a co-production, this process was informed by autistic experiences and viewpoints.

A strength, but also a limitation, was the extent of the questionnaire. Many other questions could have been used in the knowledge section of the questionnaire, to further explore this area. However, this research only used questions deemed most important as reported by members of the autistic/autism communities in Australia. The target duration of the survey was a maximum of 10 minutes. Additional questions would have increased the time taken to complete they survey, likely resulting in a poorer number of responses. Nevertheless, the demographics section of the questionnaire could have included questions about socioeconomic status, such as annual income or post code as an indicator. This could be done in future research exploring other variables that may influence attitudes and knowledge towards autism.

## Conclusion

Neurotypicals generally had high knowledge about autism. Almost all had positive attitudes towards autism. *Knowledge about Societal Views and Ideas*, and *What it Could be Like to Have Autism* positively impacted attitudes. Knowing and spending time around someone with autism further added to positive attitudes. Women had more positive attitudes than men, whereas neither age nor education level had an impact. Thus, interventions targeted to increase knowledge about and experience with autism could improve attitudes and increase acceptance of the autistic community, ultimately improving their overall quality of life.
